# A Multimodal Approach to the Quantification of Kinetic Tremor in Parkinson’s Disease

**DOI:** 10.3390/s20010184

**Published:** 2019-12-28

**Authors:** Mateusz Szumilas, Krzysztof Lewenstein, Elżbieta Ślubowska, Stanisław Szlufik, Dariusz Koziorowski

**Affiliations:** 1 Institute of Metrology and Biomedical Engineering, Faculty of Mechatronics, Warsaw University of Technology, A. Boboli 8 St., 02-525 Warsaw, Poland; 2 Department of Neurology, Faculty of Health Science, Medical University of Warsaw, Żwirki i Wigury 61 St., 02-091 Warsaw, Poland

**Keywords:** Parkinson’s disease, kinetic tremor, digitizing tablet, echo state network, machine learning

## Abstract

Parkinson’s disease results in motor impairment that deteriorates patients’ quality of life. One of the symptoms negatively interfering with daily activities is kinetic tremor which should be measured to monitor the outcome of therapy. A new instrumented method of quantification of the kinetic tremor is proposed, based on the analysis of circles drawn on a digitizing tablet by a patient. The aim of this approach is to obtain a tremor scoring equivalent to that performed by trained clinicians. Models are trained with the least absolute shrinkage and selection operator (LASSO) method to predict the tremor scores on the basis of the parameters computed from the patients’ drawings. Signal parametrization is derived from both expert knowledge and the response of an artificial neural network to the raw data, thus the approach was named multimodal. The fitted models are eventually combined into model ensembles that provide aggregated scores of the kinetic tremor captured in the drawings. The method was verified with a set of clinical data acquired from 64 Parkinson’s disease patients. Automated and objective quantification of the kinetic tremor with the presented approach yielded promising results, as the Pearson’s correlations between the visual ratings of tremor and the model predictions ranged from 0.839 to 0.890 in the best-performing models.

## 1. Introduction

One of the Parkinson’s disease (PD) symptoms related to the motor function is kinetic tremor, i.e., tremor occurring during voluntary movement [1]. Even though this tremor is not considered crucial for diagnosis, which is based on the presence of other motor manifestations including bradykinesia, rigidity, and rest tremor [2,3], it significantly affects patients’ quality of life [4]. The frequency of the kinetic tremor is reported to remain within 4–9 Hz, reaching higher than the frequency of the rest tremor (4 to 6 Hz) and lower than physiological tremor (8 to 12 Hz) [1,4,5]. Additionally, it is a highly intermittent symptom [5].

In a clinical setting, the condition of a patient suffering from PD is typically determined with clinical scales, mainly the Unified Parkinson’s Disease Rating Scale (UPDRS) [6,7]. Such assessments performed by medical personnel are subject to human-dependent variability, which impose a noise-like component on the scores. Specifically, it reduces applicability of the ratings performed by humans in case of monitoring the disease progression in its early stages, when changes in motor function are less evident [7]. Development of the instrumented methods of motor function quantification follows the need for reduction of subjectivity and improvement of resolution in relation to the existing scales. Moreover, reliable monitoring of the disease from its onset is necessary to provide feedback for an early neuroprotective therapy, if such is introduced.

In this study, the kinetic tremor is recorded with a digitizing tablet during a simple task of continuously drawing circles along a given path. The quantification is performed by models that utilize two classes of parameters, specifically: (1) parameters computed directly from the acquired signals, based on the knowledge of disease symptoms, and (2) parameters computed from the activations of echo state networks (ESN) (a class of artificial neural networks (ANN)), collected after passing the acquired trajectories through a set of ESNs.

Teaching predictive models based on both of the aforementioned groups of parameters, being a multimodal approach, is a novelty in the field of the kinetic tremor diagnostics. The solutions found in literature are based exclusively on one of two types of parameters mentioned above, commonly with a disease diagnosis or a disease stage classification as an output [8,9,10,11,12,13,14].

As for the quantification of kinetic tremor, two of the cited studies are relevant. Lin et al. [12] presented a system for assessment of the tremor severity through a digital analysis of spiral drawings by PD and essential tremor (ET) patients. The quantification was based on signal-derived parameters (no processing with ANNs was performed), and predictions correlated well with visual ratings provided by physicians. In the second study, comparably good results were reported by Legrand et al. [8], where the authors recreated visual ratings of ET patients’ tremors (according to the Bain and Findley rating scale) from seven independent raters, employing the analysis of spiral drawings in time and frequency domains.

An interesting example of parametrizing data from a digitizing tablet by features based on field-specific knowledge can be found in the study by Lopez-de-Ipna et al. [13] that was targeted on early ET diagnosis. The authors used linear and nonlinear features (the latter being complexity metrics, namely a Shannon entropy and a fractal dimension) of the drawings of spirals as an input for the classifiers such as support vector machine, multilayer perceptron neural network (MLP), and k-nearest neighbors algorithm. The achieved classification accuracies were reported to be the greatest for the MLP classifiers. It should be noted that this neural network has not been used as a source of signal parameterization, whereas in our approach it serves as such.

The two following studies showed applicability of ESN architecture for diagnosis of PD. Gallicchio et al. [11] presented an approach in which time series acquired during drawing of spirals were analyzed with deep ESNs without the signal preprocessing and feature extraction steps. Another attempt of employing ESNs to analyze and classify minimally processed signals may be found in a study by Lacy et al. [10] which addressed the problem of differentiating PD patients and control subjects. These are examples of ANN usage in which the network activations are closely related to the raw movement data, which is in line with our approach. However, ESNs served as classifiers here, while the tremor quantification is of our main interest.

The aim of our study is to look for a synergic effect in a combination of (1) a classical signal processing and (2) a signal exploration with an artificial neural network, in the task of the quantification of kinetic tremor. Additionally, only the models that provide continuous tremor scoring are considered, as any discretization of response may potentially hinder monitoring of the disease progression in its early stage.

## 2. Materials and Methods

### 2.1. Measurement System

The measurement system consists of a digitizing tablet (Intuos 2, XD-0608-U, Wacom, Vancouver, WA, USA; resolution: 0.01 mm, sampling: 100 samples/s, accuracy: ±0.25 mm, levels of pressure: 1024, upper cut-off frequency (−3 dB): 13 Hz—derived from the tablet frequency response which was measured by drawing circles along the diagonal of the active area with frequencies ranging from 1 to 25 Hz, with a motorized, rotating pen holder) connected to a personal computer running custom acquisition software [15]. Two-dimensional pen trajectory and pen pressure time series were recorded during the measurement. The acquired data were analyzed in LabVIEW 2014 (National Instruments, Austin, TX, USA) and R environment (R Base ver. 3.4.4, Vienna, Austria) [16].

During the examination, a patient traced along the given template of a circle (130 mm in diameter), centered over the tablet active area. There was no preferred direction of drawing and the drawing speed was roughly specified as “moderate”. Each examination took approximately 100 s. The patient performed the task while seated and with their elbow of the examined arm raised from the table and unsupported. The examination was carried out for both hands.

**Figure 1:**
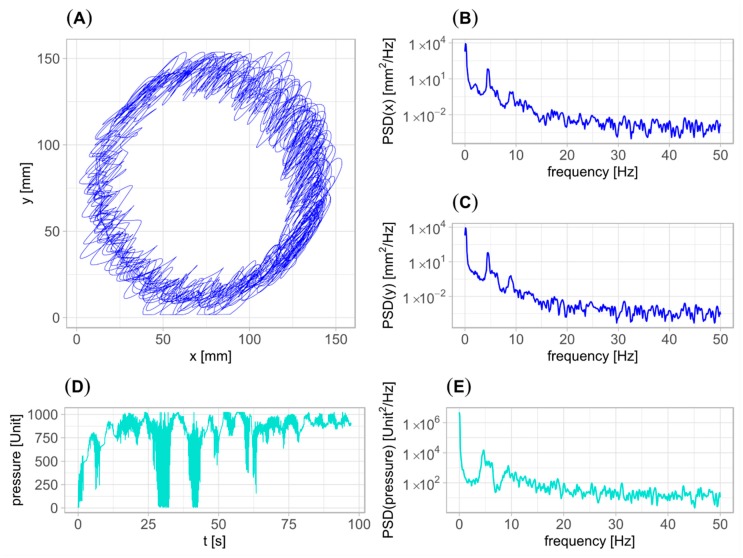
Exemplary signals acquired from the patient having a severe tremor. (**A**) two-dimensional pen trajectory; (**B**,**C**) power spectral densities (PSDs) of *x* and *y* pen trajectory coordinates, respectively; (**D**) pen pressure. (**E**) PSD of pen pressure.

Exemplary signals acquired from the patient having severe tremor (rated 68 points in UPDRS part III—motor examination) are presented in Figure 1 along with respective power spectral densities (estimated with the Welch’s method; parameters: 1024 frequency bins, Hann window of 1000 samples, 80% overlap). A spectral peak related to the patient’s tremor is seen between 4 and 5 Hz for each signal. The large-amplitude artifacts, present between 25 and 50 s of the pen pressure time series, arise due to accidentally drawing outside the tablet active area.

### 2.2. Data

The measurements were taken from 64 PD patients, of which 30 were females (mean age ± standard deviation (SD): 55.8 ± 10.1) and 34 were males (mean age ± SD: 57.9 ± 10.4). The patients underwent a surgical procedure (implantation of deep brain stimulation) and/or were treated with pharmacotherapy. In this study, however, the type of treatment was not considered as a differentiating factor.

The examinations were carried out in the Department of Neurology of the Medical University of Warsaw (research was under approval of the Bioethics Committee of the Medical University of Warsaw, all subjects gave written informed consent in accordance with the Declaration of Helsinki). The patients were repeatedly examined by trained clinicians during subsequent appointments, which resulted in 1100 recorded drawings accompanied by UPDRS ratings. The item 21 from the UPDRS scale (UPDRS.21) was of special interest for this study, as it is meant for scoring of the kinetic or postural tremor of hands.

The kinetic tremor in each recording was rated with a proposed scale, named *tremor_score*. The *tremor_score* is based on the visual analysis of a 2D trajectory and a power spectrum of the recorded drawing. The score is assigned from 0 (“no tremor”) to 10 (“severe tremor”) on an integer, monotonic scale. To improve consistency of ratings, these should be performed in uninterrupted sessions with subsets of data as large as possible. The rating person does not accompany patients during their examinations, nor do they have any additional information about them, such as duration of the disease or assigned UPDRS scores.

The *tremor_score* is designed as a modification of the UPDRS.21, with the following differences: only the kinetic tremor is considered (specifically, scoring of the postural tremor is avoided), the range of scores is extended and assumed as strictly monotonic (the UPDRS.21 scores range from ‘0’ to ‘4’ and scores ‘2’ and ‘3’ differ only in the presence of the postural tremor, while both correspond to the kinetic tremor of “moderate” amplitude; as such, the UPDRS.21 scores are weakly monotonic in relation to the kinetic tremor), and, finally, the rating person has insight in the power spectrum of the signal in order to support scoring of small-intensity tremors.

We note that the *tremor_score* differs from the widely adopted scale of tremor severity by Bain and Findley [17] in that it: (1) encompasses visual analysis of the signal power spectrum and (2) enables the rating person to subset the recording and to plot the original trajectory only partially. We consider the latter especially important, as in the case of circle-drawing task, the circles drawn by a patient in subsequent repetitions overlay and intersect, which can obscure manifestations of tremor.

### 2.3. Spectrum-Based Tremor Quantification

Occurrence of a tremor component in a signal manifests itself in a form of prominent peaks in its power spectrum. A method of quantification of such a tremor manifestation is proposed, yielding a single parameter *tQ*, being a ratio of a tremor-related signal power and an averaged background signal power. The *tQ* parameter is obtained from the traced trajectory (stored in the form of time series of Cartesian coordinates {***x***(*t*), ***y***(*t*)}) in the following steps:

1. The acquired time series {***x***(*t*), ***y***(*t*)} are high-pass filtered with 0.5 Hz cut-off frequency and transformed with short-time Fourier transform (STFT) configured as follows: Hann window of 512 samples, 5-sample step (0.05 s), 256 frequency bins.
2. Combined spectrogram STFTc(f,t) is computed as a sum of squared STFTs of ***x***(*t*) and ***y***(*t*) according to Equation (1):
  (1) $$STFTc\left(f,t\right)=\;{\left|STFTx\left(f,t\right)\right|}^{2}+{\left|STFTy\left(f,t\right)\right|}^{2}\;.$$
3. The STFTc(f,t) spectrogram is divided into 2-s-long segments with 0.05 s step. For each segment of the spectrogram, a relevant power spectral density (PSD) denoted by $S_{i}\left(f\right)$ is computed.
4. We have observed that a common feature of the obtained $S_{i}\left(f\right)$ is the presence of a relatively large spectral component with the power density reciprocally related to the frequency. It can be written in the form $S_{Ri}\left(f\right)\propto {\left|f\right|}^{-\alpha }$, where *α* takes values greater than 0. $S_{Ri}\left(f\right)$ may be attributed to the spectral leakage from a large amplitude and low frequency signal of circle tracing, an artefact of the filtering from step 1. This component should be removed because it hinders the detection of tremor-related peaks. To remove $S_{Ri}\left(f\right)$ from $S_{i}\left(f\right)$, a function of the form $S_{Ni}\left(f\right)=b_{i}{\left|f\right|}^{-\alpha_{i}}$ is fitted to the spectrum in the range 2–26 Hz (fitting is performed in logarithmic coordinates, using the method of least absolute residuals, as this method shows insensitivity to large outliers [18], here being the spectral peaks). The $S_{i}\left(f\right)$ is divided by the fitted function $S_{Ni}\left(f\right)$, yielding the residual spectral density $S_{Ti}\left(f\right)$.
5. $S_{Ti}\left(f\right)$ is divided into 26 frequency bins that range from 2 to 15 Hz, each having the width of 0.5 Hz. For every bin, the signal variance (power) is computed, denoted by $\sigma_{i,k}^{2}$ for *k*-th bin. The mean frequency of the bin with the greatest signal variance is denoted by $f_{tQ,i}$.
6. Variances related to the *i*-th data segment are sorted in ascending order, thus forming a 26-element set *W_i_*, where *k*-th variance is denoted by $W_{i,k}^{\;}$. The corresponding parameter *tQ_i_* is then computed according to Equation (2). Variances $W_{i,25}^{\;}\;$and $W_{i,24}^{\;}$ are not averaged in the denominator of the equation. It is assumed that these may be related to $W_{i,26}^{\;}$ by conveying the power of signal harmonics, hence they should not be considered for background power estimation:
  (2) $$tQ_{i}=\frac{W_{i,26}}{\frac{1}{23}\sum_{j=1}^{j=23}W_{i,j}}.$$

### 2.4. Power Spectral Density and Signal Entropy

In addition to the computation of the *tQ* parameter, the power of the signal in the band above 3 Hz is computed. Hence, a parameter named *PSDc_var_3Hz* is obtained based on the total PSD, being a sum of partial PSDs of ***x***(*t*) and ***y***(*t*). The PSD is computed with the Welch’s method (1024 frequency bins, Hann window of 1000 samples, 80% overlap).

As the analyzed signals represent activity of a complex system, we decided to include a measure of their complexity in the parameters set. We refer to the sample entropy (SampEn) [19], defined as the negative natural logarithm of the conditional probability that two distinct sequences of *m* samples that are similar (i.e., the distance between them is smaller than the threshold *r*, where the distance is measured with the Chebyshev metric) will remain similar after extending them with the next samples (i.e., to the length *m*+1). To analyze the signal in different time scales, a multiscale entropy (MSEn) method can be applied [20], in which the computation of the SampEn is preceded by averaging and decimation of the original signal {*x*_1_,..., *x_i_*,..., *x_N_*}. The samples of the resulting coarse-grained signal $y_{\;}^{\left(\tau \right)}$ are calculated, according to the equation [20]:

(3) $$y_{j}^{\left(\tau \right)}=\frac{1}{\tau }\sum_{i=\left(j-1\right)\tau +1}^{i=j\tau }x_{i},\;\;\;\;\;\;\;\;\;\;\;\;\;1\leq j\leq N/\tau ,$$

where: *τ* is decimation (scale) coefficient, *x_i_* is *i*-th sample of the original signal, and *N* is the length of the original signal.

The MSEn is calculated for the drawing velocity component (denoted by *V_t_*) that is transverse to the reference circle. Long-term trends are removed from the signal during preprocessing when the signal is high-pass filtered over 0.05 Hz (a finite impulse response (FIR) filter with a Hann window of 2000 samples).

The set of 19 parameters derived from the time series was extended with log-transformations of the parameters that had the most skewed distributions in randomly sampled data subsets. The skewness was computed from 100 randomly sampled subsets, each consisting of 60% of the complete dataset, and the parameters which average skewness minus one standard deviation was greater than 3 were transformed with base 10 logarithm (4 parameters met this condition). The resulting 23 parameters are summarized in Table 1.

### 2.5. Echo State Network Architecture

An echo state network is a type of recurrent neural network (RNN), in a classical approach characterized by a randomly generated hidden layer with untrained connections, where only the output weights are subject to supervised training [21,22]. Such a hidden layer is called *reservoir* [23], and it is able to memorize the time series fed to the network. The reservoir should satisfy the echo state property, i.e., the state of the reservoir should be uniquely defined by the fading history of the input signal. Echo state property is ensured by the proper adjustment of the network hyperparameters, which are parameters governing the distribution of connection weights.

In this work, we assume the following update equation of the ESN with *N* neurons in the reservoir [22]:

(4) $$x\left(t\right)=\left(1-\alpha \right)\;x\left(t-1\right)+\;\alpha \tanh\left(W_{in}\left[1;u\left(t\right)\right]+W\;x\left(t-1\right)\right),$$

where: $x\left(t\right)\in \;{\mathbb{R}}^{N}$ is a vector of reservoir activations at the time step *t*, $u\left(t\right)\in \;{\mathbb{R}}^{M\times T}$ is an *M*-dimensional input signal of length *T*, $W_{in}\left(t\right)\in \;{\mathbb{R}}^{N\times M+1}$ and $W\left(t\right)\in \;{\mathbb{R}}^{N\times N}$ are the input and reservoir weight matrices, respectively, [•;•] is the vertical concatenation operator, α∈(0,1] is the leaking rate and tanh(•) is the element-wise hyperbolic tangent function.

The ESN output is defined as:

(5) $$y\left(t\right)=W_{out}\left[1;x\left(t\right)\right],$$

where: $y\left(t\right)\in \;{\mathbb{R}}^{My}$ is the *M_y_*-dimensional network output and $W_{out}\in \;{\mathbb{R}}^{My\times N+1}$ is the output weight matrix.

**Figure 2:**
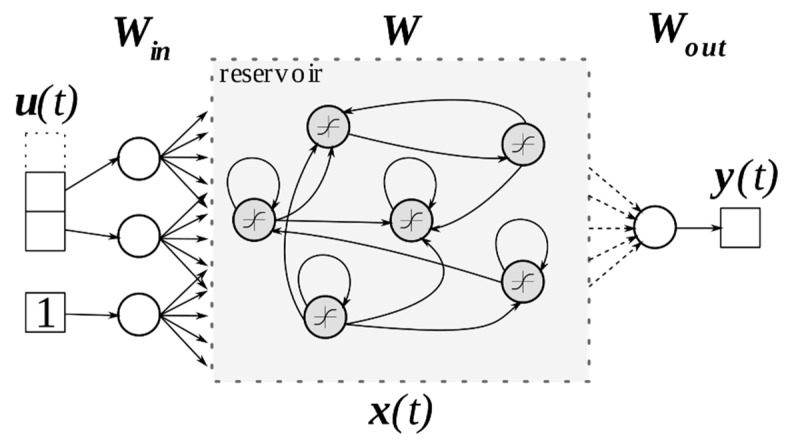
The schematic of echo state network (ESN) architecture. Groups of arrows directed from/to the reservoir indicate connections to all neurons. Solid lines denote fixed weights, whereas dashed lines denote weights computed during training using linear regression. Names of vectors and matrices are situated next to corresponding network components.

A schematic illustration of the ESN architecture is presented in Figure 2.

The reservoir matrix W and input weights $W_{in}\;$are initialized randomly from uniform distributions over [−0.5;0.5] and [−0.5ω;0.5ω], respectively, where *ω* is an input scaling parameter. The matrix ***W*** is typically generated sparse, with sparsity (i.e., proportion of non-zero elements in matrix) denoted by *s_W_*.

An important hyperparameter of the network is a spectral radius ρ(W) of the reservoir, i.e., the maximum absolute eigenvalue of W. When network is configured with leaky integration in nodes (α≤1), the effective spectral radius has to be calculated according to the equation [24]:

(6) $$\rho \left(\tilde{W}\right)=\rho \left(\alpha W+\left(1-\alpha I\right)\right)\;,$$

where $I\in {\mathbb{R}}^{N\times N}$ is an identity matrix. In most cases, setting $\rho \left(\tilde{W}\right)<1$ is sufficient to ensure the echo state property. The spectral radius of matrix is adjusted in two steps: first, the matrix is element-wise divided by its current spectral radius, and, second, it is element-wise multiplied by the value of the desired spectral radius.

The ESN shows increased processing capability in the state between ordered and chaotic dynamics, that is at the *edge of chaos* [25,26]. The stability of dynamical system may be estimated by its largest Lyapunov exponent, i.e., a measure of divergence (in the state space) of the system trajectories with infinitesimally small initial separation, as defined in Equation (7) [25]:

(7) $$\lambda_{max}=\;\underset{k\to \infty }{\lim}\frac{1}{k}\ln\left(\frac{\gamma_{k}}{\gamma_{0}}\right),$$

where: $\gamma_{0}$ is the initial distance between the two considered trajectories and $\gamma_{k}$ is the distance at time *k*.

The value of $\lambda_{max}<0$ is characteristic for stable systems, while $\lambda_{max}>0$ indicates that the system shows chaotic behaviour. The phase transition occurs at the aforementioned *edge of chaos* for $\lambda_{max}\approx \;0$. As the largest Lyapunov exponent is defined asymptotically, it has to be estimated (we denote the estimated exponent by $\hat{\lambda }$). In this work, we use method described in [25], where it was adopted from the more generic approach found in [27].

Moreover, we propose and implement a complementary method of adjusting $\hat{\lambda }$ of a network according to the iterative procedure presented in Algorithm 1, founded on the assumption of monotonic relation between$\;\rho \left(\tilde{W}\right)$ and $\hat{\lambda }$. The algorithm does not ensure that $\hat{\lambda }$ is set with assumed tolerance $\Delta \lambda_{0}$, as the closest reached solution is returned. If the result of the adjustment procedure is not acceptable, one should reinitialize W matrix and rerun the procedure.
**Algorithm 1**. Lyapunov exponent adjustment procedure1:
**procedure**Adjust_Lyapunov_Exponent ($\tilde{W}$, Δρ, $\lambda_{0}$, $\Delta \lambda_{0},$$N_{\lambda }$)2:
 **for** i: = 1 **to** $N_{\lambda }$ **step by** 1 **do**3:
  $\hat{\lambda }\left[i\right]$: = Estimate_Lyapunov_Exponent ($\tilde{W}$);4:
  $\tilde{W}\left[i\right]$: = $\tilde{W}$;5:
  $\Delta \lambda {\left[i\right]}_{\;}$: = Absolute_Value ($\hat{\lambda }\left[i\right]-\lambda_{0}$);6:
  //stop if: estimated value is within the tolerance range7:
  //or does not change between iterations8:
  **if** ($\Delta \lambda {\left[i\right]}_{\;}\leq \;\Delta \lambda_{0}$) OR (Δλ[i]==Δλ[i−1]) **then**9:
   **break for** i **loop**;10:
  **end if**11:
  **if** (Sign (Δλ[i])! = Sign (Δλ[i−1])) **then**12:
   Δρ: = Δρ/2;13:
  **end if**14:
  $\rho_{tmp\;}:=\;$Compute_Spectral_Radius ($\tilde{W}$);15:
  //adjust $\tilde{W}$ spectral radius to $\rho_{tmp\;}\pm \Delta \rho$16:
  **if** (Sign (Δλ[i]) >0) **then**17:
   $\tilde{W}:=\;$Set_Spectral_Radius ($\tilde{W},\;\rho_{tmp\;}-\Delta \rho$);18:
  **else**19:
   $\tilde{W}:=\;$Set_Spectral_Radius ($\tilde{W},\;\rho_{tmp\;}+\Delta \rho$);20:
  **end if**21:
 **end for** i22:
 i_out: = Index_of_Minimum_Value (Δλ[]);23:
 return { $\tilde{W}\left[i\_\mathrm{out}\right],\;\;\hat{\lambda }\left[i\_\mathrm{out}\right]$};  **end procedure**

The recurrent structure of the ESN enables memorization of input sequences. The measure of network short-term memory is called memory capacity (MC) and is estimated through teaching the ESN to recover its past inputs. The MC is calculated according to [25], with uniformly random time series from the interval [−1; 1] used as an input.

### 2.6. ESN Input Preprocessing

Acquired trajectories {***x***(*t*), ***y***(*t*)} were preprocessed before being used as an ESN input ***u***(*t*), according to the following procedure:

1. Components ***x***(*t*) and ***y***(*t*) were bandpass filtered (FIR filter with 1–12 Hz passband, Hann window of 1000 samples) and decimated with factor 2.
2. Principal component analysis (PCA) was performed on the filtered data and the first component *PC*_1_ was retained for further computations.
3. To normalize the data, the *PC*_1_ component was divided by its interquartile range (IQR).

### 2.7. Parameters Based on the ESN Activation

States of the ESN for *i*-th processed trajectory are combined as columns, yielding an activation matrix $S_{i}\in {\mathbb{R}}^{N\times T}$. Each activation matrix is summarized by a vector of parameters, for $S_{i}$ defined as follows:

(8) $$d_{i}=\;\left\{x_{i}^{rms},\;x_{i}^{sd},w_{out}^{i@+1},w_{out}^{i@-MC/2}\;\right\},$$

where:

(9) $$x_{i}^{rms}=\;\sqrt{\frac{1}{T}\overset{T}{\underset{j=0}{\sum }}S_{i}{\left[:,j\right]}^{2}}$$

is the vector of time-averaged activations of neurons (operator [:, *j*] denotes extraction of the *j*-th column of a matrix) and

(10) $$x_{i}^{sd}=\;\sqrt{\frac{1}{T-1}\overset{T}{\underset{j=0}{\sum }}{\left(S_{i}\left[:,j\right]-\frac{1}{T}\overset{T}{\underset{k=0}{\sum }}S_{i}\left[:,k\right]\right)}^{2}}\;$$

is the vector of standard deviations of activations of neurons.

Vectors $w_{out}^{i@+1}\;\epsilon \;{\mathbb{R}}^{N}$ and $w_{out}^{i@-MC/2}\;\epsilon \;{\mathbb{R}}^{N}$ are the output weights of ESN trained for prediction (one step ahead, thus superscript “i@+1”) and reconstruction of delayed signal (MC/2 steps back, thus superscript “i@−MC/2”), respectively. In both cases, the output layer is trained using linear regression and the bias component is removed from the obtained vectors of weights. To embed the information about the original signal amplitude in the weights, these are calculated for the task of prediction/reconstruction of the unnormalized signal, that is, the component *PC*_1_ is not divided by its IQR when used as a target during training (see Section 2.6).

### 2.8. Score Prediction Model

The parameters computed from the complete dataset are merged into matrices which are used to train and test models of the *tremor_score* and UPDRS.21 scales. Three matrices of parameters are considered: **D_TS_**—a matrix of 23 parameters retrieved from time series, **D_ESN_**—a matrix of 4*N* parameters based on ESN structure and activation, **D_ESN+TS_**—a row-wise combination of **D_TS_**, and **D_ESN_** matrices. Each matrix is split into two submatrices for training and testing of the models, which consist of 60% and 40% of the original matrix, respectively. The split is performed by stratified random sampling to keep proportion of scores from the complete dataset. For each scale, the following models are considered:

(11) $$M_{TS}:\hat{y_{TS}}=g\left(f_{TS}\left(D_{TS}\right)\right),$$

(12) $$M_{ESN}:\;\hat{y_{ESN}}=g\left(f_{ESN}\left(D_{ESN}\right)\right)\;,$$

(13) $$M_{ESN+TS}:\hat{\;y_{ESN+TS}}=g\left(f_{ESN+TS}\left(D_{ESN+TS}\right)\right),$$

(14) $$g\left(y\right)=\{\begin{array}{cc} y_{max}+0.5\tanh\left(y-y_{max}\right), & if\;y>y_{max}\\ y_{min}+0.5\tanh\left(y-y_{min}\right), & if\;y<y_{min}\\ y, & otherwise \end{array},$$

where: $f_{\ldots }\left(•\right)$ is a linear function of parameters, fitted to predict the score of interest from the parameters matrix $D_{\ldots }$**,** $\hat{y_{\ldots }}$ is a prediction of the score, g(•) is a limiting function as defined in (14), {$y_{max}$, $y_{min}$} are the maximum and minimum values of the modelled scales, that is, {10, 0} and {4, 0} for the *tremor_score* and UPDRS.21, respectively.

The $f_{\ldots }\left(•\right)$ functions are fitted using the least absolute shrinkage and selection operator (LASSO) method [28]. The LASSO method performs variable selection and regularization, which is considered especially important in the case of high-dimensional training sets that include ESN-derived parameters. The LASSO output depends on a regularization parameter *λ*_LASSO_ chosen with 10-time, 5-fold cross-validation. Two values of cross-validated *λ*_LASSO_ are considered, that is, *λ_MIN_* and *λ*_1*SE*_. The parameter *λ_MIN_* corresponds to the models with the minimal mean squared error (MSE) of prediction. The parameter *λ*_1*SE*_ corresponds to the models with the minimal number of non-zero coefficients and prediction MSE not greater than the minimal MSE computed during cross-validation, increased by its standard error [29].

Due to the random initialization of the ESNs, the models that are based on their activations ($M_{ESN}\;$and $M_{ESN+TS}$) show increased variability of prediction quality when compared with the models that use only parameters computed from the time series ($M_{TS}$). To reduce the effect of initialization randomness on the prediction, an ensemble of models is established [30,31]. The ensemble is formed by combining ESN-based predictors (with hyperparameters as presented in Table 2), and the aggregated output is computed as an average of the ensemble models’ predictions. In total, the ensemble consists of 216 individual models. To analyze the variability of the aggregated ensemble response, the models were recomputed 50 times, i.e., 50 independent and randomly initialized ensembles were formed.

**Figure 3:**
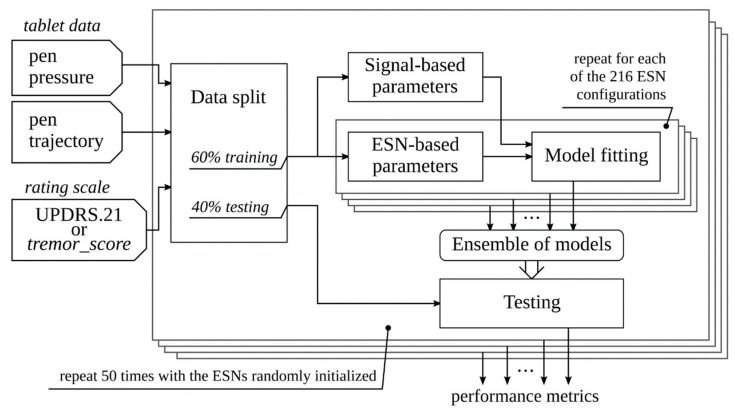
The diagram of the process of training and testing ensembles of models. The ensembles are formed and tested 50-fold. In each fold: (1) the ESNs used for computation of parameters are randomly reinitialized, and (2) the signal-based parameters are reused during model fitting while the ESN-based parameters are computed separately for each of the configurations considered.

The diagram summarizing the process of training and testing models is presented in Figure 3.

## 3. Results

The Pearson’s correlation coefficients between the signal-based parameters and the target scales are computed for the complete dataset and shown in Table 3. If correlations having absolute value greater than 0.1 are considered, then these are consistently higher for the *tremor_score* than for the UPDRS.21 scale. The log-transformed parameters *PSDc_var_3Hz_log,* *avg_tQ_max_log* and *sd_tQ_max_log* show higher absolute correlations than the respective source parameters, thus confirming the relevance of performing such a transformation. The highest absolute correlations are observed for *avg_tQ_max_log* (*r* = 0.73 for *tremor_score* and *r* = 0.52 for UPDRS.21), which supports the idea of computing *tQ* as a measure of tremor. The lowest absolute correlations are present for the parameters derived from the low-pass filtered *V*_t_. On the contrary, MSEn (τ∈{25, 50}) computed for the *V*_t_ has one of the highest absolute correlations for both target scales.

**Figure 4:**
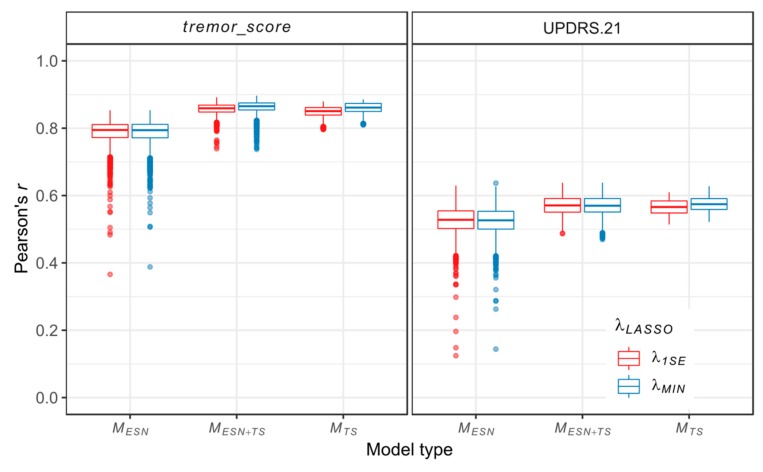
Distribution of correlations (measured with Pearson’s *r*) between individual model predictions and target values. Results are grouped according to the model type and the *λ*_LASSO_ regularization parameter.

As a metric of prediction quality of the studied models, the correlations between their responses and the target scores are computed. Since the proposed models provide continuous outputs, the prediction quality is measured with Pearson’s *r* correlation coefficient. The results for individual models are shown in Figure 4.

The highest median of Pearson’s *r* is achieved by $M_{ESN+TS}$ models for the *tremor_score* and $M_{TS}$ models for the UPDRS.21, in each case with *λ_MIN_* regularization parameter. The $M_{ESN}$ models show greater variability of prediction quality when compared with $M_{ESN+TS}$ models and $M_{TS}$ models. Especially, the $M_{TS}$ models show no (UPDRS.21) or a very limited number (*tremor_score*) of outliers, while numerous $M_{ESN}$ models have significantly lowered prediction quality (we define outliers as models with Pearson’s *r* less than the lower quartile of the *r* distribution minus 1.5 times its interquartile range (IQR) or greater than the upper quartile plus 1.5 IQR).

**Figure 5:**
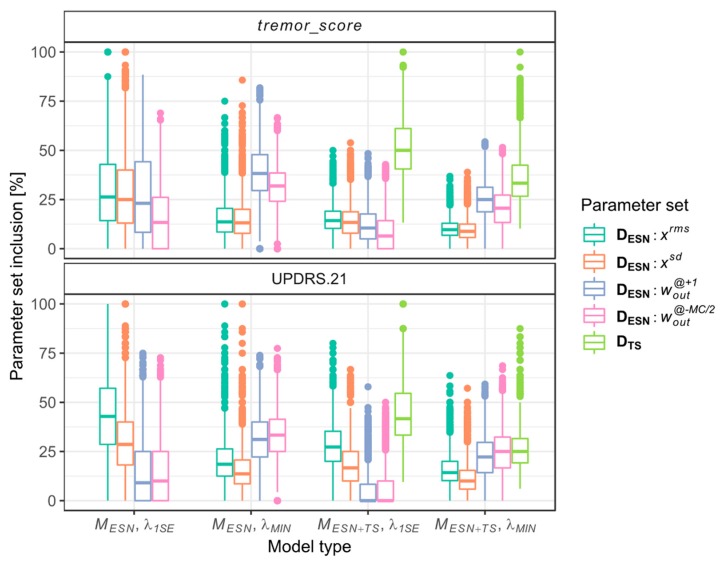
Inclusion of parameters from **D_TS_** and **D_ESN_** sets in individual $M_{ESN}$ and $M_{ESN+TS}$ models (as a percentage of model parameters). Results are grouped according to the model type and *λ*_LASSO_ regularization parameter.

The analysis of individual models provides information about general patterns of inclusion of the parameters from matrices **D_TS_** and **D_ESN_,** with the latter partitioned in subsets of parameters $x^{rms}$, $x^{sd}$, $w_{out}^{i@+1}$ and $w_{out}^{i@-MC/2}$. The results are presented in Figure 5.

In the case of *tremor_score*, the *λ*_1*SE*_-regularized models show approximately equal contributions of **D_ESN_** parameters’ subsets, while in the *λ_MIN_*-regularized models, the percentage of $w_{out}^{i@+1}$ and $w_{out}^{i@-MC/2}$ parameters is higher than $x^{rms}$ and $x^{sd}$. Similar contributions of parameters may be observed in the case of models of the UPDRS.21 scale, which differ in having greater percentage of $x^{rms}$ and $x^{sd}$ parameters at the expense of decreased percentage of $w_{out}^{i@+1}$ and $w_{out}^{i@-MC/2}$ parameters.

**Figure 6:**
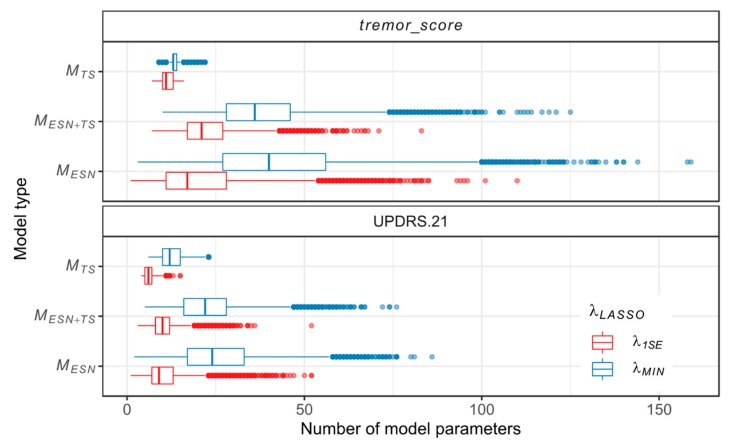
Total parameter counts of individual models, grouped according to the model type and *λ*_LASSO_ regularization parameter.

For both scales and with both types of regularization, models of $M_{ESN+TS}$ type are dominated by **D_TS_** parameters, as their median count is greater than the respective median counts from the remaining groups of parameters. Inclusion of **D_TS_** parameters does not lead to a consistent change in the number of $M_{ESN+TS}$ model parameters when compared with $M_{ESN}$ models. However, it reduces the spread of the parameter count (quantified by IQR), which is shown in Figure 6.

**Figure 7:**
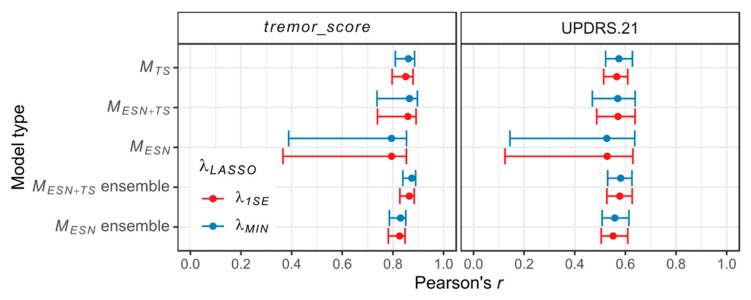
Prediction quality of different types of models, as achieved with the test set. The median correlation for each model type is marked by a dot, the whiskers extend between the minimum and maximum values of correlation.

The results for model ensembles and individual models are shown in Figure 7 and Table 4. The highest correlations of predictions and target scores are observed in the case of individual $M_{ESN+TS}$ models. The maximum Pearson’s *r* of $M_{ESN+TS}$ models is equal to 0.638 (UPDRS.21, *λ_MIN_* and *λ*_1*SE*_), 0.897 (*tremor_score*, *λ_MIN_*) and 0.891 (*tremor_score*, *λ*_1*SE*_). Adoption of the ensemble approach leads to reduction of the maximum prediction quality in the case of best $M_{ESN+TS}$ ensembles as compared with the best individual $M_{ESN+TS}$ models (maximum values: *r* = 0.626 for UPDRS.21 (*λ_MIN_* and *λ*_1*SE*_), *r* = 0.890 for *tremor_score* (*λ_MIN_*) and *r* = 0.884 for *tremor_score* (*λ*_1*SE*_)). Nevertheless, it does essentially improve outcome in the worst-case scenario, as the $M_{ESN+TS}$ ensembles outperform models of other types with respect to the minimum prediction quality (minimum values: *r* = 0.530 for UPDRS.21 (*λ_MIN_*), *r* = 0.527 for UPDRS.21 (*λ*_1*SE*_), *r* = 0.839 for *tremor_score* (*λ_MIN_*) and *r* = 0.827 for *tremor_score* (*λ*_1*SE*_)).

## 4. Discussion

Automated quantification of the kinetic tremor with the presented multimodal approach provides best results in the case of predictive models based on the *tremor_score* scale. These models achieve maximum correlation between the predictions and original scores equal to 0.897 in the test set. Results of the UPDRS.21 modelling are inferior, as the highest Pearson’s *r* for this scale is equal to 0.639.

Such a difference between outcomes for the two analysed scales originates from differences in their design. Whereas the *tremor_score* is dedicated solely for the quantification of kinetic tremor, the UPDRS.21 scale combines additional information about the postural tremor. During the circle-drawing task, the postural tremor may be recorded only if it propagates through the patient’s body from different body segments. However, possibility of the postural tremor emergence during the examination is minimized, as the patient is seated and all of their not supported body parts, excluding a head, are involved in the drawing action. Therefore, while the presence of postural tremor during the UPDRS.21 examination changes the assigned score, it does not manifest itself in the recorded data and consequently increases the prediction error. Moreover, even observation of kinetic tremor during the aforementioned examination does not imply that a tremor with the same characteristics will be present in the course of circle-drawing task, as the kinetic tremor is an intermittent symptom.

Parameters computed directly from the time series have a significant impact on the quality of prediction. The $M_{TS}$ models, which are based exclusively on the **D_TS_** parameter set, show the lowest variance of prediction quality from all three types of individual models. Comparable variances are achieved with ensemble models, however at the expense of considerable growth of the parameter set. The rationale of this result is that the data-derived parameters are chosen with some field-specific knowledge about the quantified phenomenon, while the network-derived parameters are related to this phenomenon only indirectly, and as such have to be introduced to the model in greater numbers to compensate for their potentially weak predictive power.

We compare our results related to the *tremor_score* models to the results found in recent studies aimed at evaluation of the kinetic tremor with a digitizing tablet, where (1) a type of visual rating score (VRS) of spiral drawings was used as a reference and (2) proposed models had a linear output suitable for evaluation of its correlation with VRS.

We refer to two studies, the first being the work by Lin et al. [12], where the authors reported Pearson’s correlations between the VRS and the investigated signal-derived parameters to be as high as 0.973. The models of VRS were trained and validated with digitized spiral drawings from ET and PD patients. Specifically, the training and test datasets consisted of approximately 140 and 30 recordings, respectively. In the second work, Legrand et al. [8] computed four time- and frequency-domain parameters using 92 spiral drawings acquired from 13 ET patients (the spirals were drawn with the dominant hand). The complete dataset was used for validation, as no model training was necessary. The resulting Pearson’s correlations of parameters and VRS were between 0.79 and 0.87. Nevertheless, the authors note that from the proposed parameters that only the one showing *r* = 0.82 is suitable for analysis of drawings other than spirals.

As the best *tremor_score* model ensembles proposed in our study were characterized by the Pearson’s *r* between 0.839 and 0.890, we state that our approach provides performance similar to these found in the referenced works. However, direct comparison of results is not appropriate, as there are some notable differences in the referenced methods, namely: (1) combining the data from ET and PD patients, (2) employing a spiral-drawing task and (3) being tested on significantly smaller datasets.

There is a limited number of works regarding modelling of the UPDRS.21 score based on the PD patients’ digitized drawings. In the most relevant study, Saunders-Pullman et al. [32] selected spiral-related parameters showed Spearman’s correlations between 0.40 and 0.24 with the combined UPDRS.21 score (a sum of scores of both arms). In addition, 100% of the dataset (74 PD patients, 10 spirals collected for each hand) was used to fit linear models and compute correlations. As in the case of *tremor_score*, we refrain from performing strict comparison of the aforementioned results with our findings, as the former were achieved for a spiral-drawing task and the Spearman’s rather than Pearson’s correlations were computed as a performance metric. However, we hypothesize that these linear models may be outperformed by the best of the UPDRS.21 model ensembles from our work, for which we have computed Pearson’s *r* ranging from 0.530–0.626.

It is an open research question whether our approach would provide results of a similar quality if the data from ET patients were to be quantified. We recognize this generalizability issue as very important in the context of targeting the kinetic tremor, as this symptom is widespread in ET. Combining data from PD and ET patients would allow for better comparison with, e.g., work by Lin et al. [12]. However, one should be aware that, during the model fitting, such a combination may result in discarding parameters that are specific for only one of the diseases, yielding models that are more general, albeit with inferior prediction quality when tested solely in PD or ET populations.

Future research should include testing of the repeatability of the scores obtained with the proposed models. It would be beneficial to analyze how the predictions change when the models are trained with ratings from different raters. Moreover, before our method can be applied in its target environment in the form of clinically-usable models, it is necessary to prepare a refined dataset for their training. The main difficulty in establishing such a dataset is to ensure balanced inclusion of the samples representing tremors of different intensities, as in the PD patients the most severe tremors are less frequent than those of moderate severity.

Some of the possible applications of our method are in the following two areas of PD management: (1) tremor scoring when a patient is far from a medical facility and (2) providing additional feedback for a clinician who performs adjustments of deep brain stimulation (DBS) parameters. Tremor scoring in the absence of a trained clinician can be a useful tool supporting medical personnel in the case of teleconsultations. It also enables PD patients to supplement their self-reports with objective ratings of the kinetic tremor, e.g., allowing patients to record it exactly when it appears to be most prominent. As for the second possibility, increasing repeatability of the tremor assessment during the DBS programming is a factor supporting automation of this time-consuming process. The tremors of PD respond quickly to the changes in stimulation settings [33]; therefore, their ratings provide information relevant for tuning the DBS parameters.

## 5. Conclusions

The method presented in this study is based on a widely adopted idea of analysing kinetic tremor recorded with a digitizing tablet during tasks involving drawing or writing. Our main contribution is showing that the kinetic tremor can be effectively quantified with a method, which benefits from merging two modes of data parametrization: first, the parametrization derived from the expert knowledge, and, second, the parametrization that refers to the structure of a trained echo state network and its activation in response to the data. The verification performed with clinical data showed that the models using both types of the aforementioned parametrizations outperform the ones based only on a single class of the parameters. Moreover, we combined such individual models in model ensembles. The Pearson’s correlations between the visual ratings of tremor and the model predictions in the best-performing model ensembles ranged from 0.839 to 0.890. The individual models from these ensembles showed more varied correlations between 0.737 and 0.897.

## Figures and Tables

**Table 1:** Parameters computed from the time series.

Parameter	Description
*PSDc_var_3Hz* *PSDc_var_3Hz_log*	Total power of the signal in the band above 3 Hz and its log_10_ transformation
*f_tQ_mod*	Mode of $f_{tQ,i}$ values
*abs_f_tQ_dist_5Hz*	Absolute difference of *f_tQ_mod* and 5 Hz, i.e., |*f_tQ_mod*-5|
*avg_tQ_max* *avg_tQ_max_log*	Average of *tQ* in the segments representing 10 s of measurement with the highest *tQ* values (i.e., 200 segments with highest *tQ* in the case of 0.05 s segment step) and its log_10_ transformation
*sd_tQ_max* *sd_tQ_max_log*	Standard deviation of *tQ* in the segments as used in *avg_tQ_max* computation and its log_10_ transformation
*cv_tQ_max*	Coefficient of variation: *sd_tQ_max/avg_tQ_max*
*avg_alpha*	Average of *α* of $S_{i}\left(f\right)$ component
*sd_alpha*	Standard deviation of *α* of $S_{i}\left(f\right)$ component
*cv_alpha*	Coefficient of variation: *sd_alpha/avg_alpha*
*avg_abs_Vt.LP.1*	Average of the absolute transverse velocity component of drawing motion (*V_t_*), low-pass filtered with 1 Hz cut-off frequency
*sd_abs_Vt.LP.1* *sd_abs_Vt.LP.1_log*	Standard deviation of the absolute transverse velocity component of drawing motion (*V_t_*), low-pass filtered with 1 Hz cut-off frequency and its log_10_ transformation
*cv_abs_Vt.LP.1*	Coefficient of variation: *sd_abs_Vt.LP.1/avg_abs_Vt.LP.1*
*avg_press*	Average pen pressure
*sd_press*	Standard deviation of the pen pressure
*cv_press*	Coefficient of variation: *sd_press/avg_press*
*Vt.HP.C5_MSEn_2_5*	Multiscale entropy (*m* = 2, *τ =* 5) of the high-pass filtered *V_t_* (0.05 Hz cut-off frequency)
*Vt.HP.C5_MSEn_2_10*	Multiscale entropy (*m* = 2, *τ =* 10) of the high-pass filtered *V_t_* (0.05 Hz cut-off frequency)
*Vt.HP.C5_MSEn_2_25*	Multiscale entropy (*m* = 2, *τ =* 25) of the high-pass filtered *V_t_* (0.05 Hz cut-off frequency)
*Vt.HP.C5_MSEn_2_50*	Multiscale entropy (*m* = 2, *τ =* 50) of the high-pass filtered *V_t_* (0.05 Hz cut-off frequency)

**Table 2:** Parameters of echo state networks (ESNs) used in models combined in an ensemble.

Parameter	Description	Values
*N_REP_*	Repetitions of ESNs with common hyperparameters	4
*N*	Number of ESN neurons	{50, 100, 200}
$\hat{\lambda }$	Adjusted Lyapunov exponent of the reservoir	{−0.2, −0.05} adjusted with ±0.001 tolerance
α	Leaking rate	{0.6, 0.9, 1}
ω	Input scaling	{0.1, 1, 10}
*s_W_*	Reservoir sparsity	0.1

**Table 3:** Pearson’s correlation coefficients between the signal-based parameters and the target scales.

Parameter	*tremor_score*		UPDRS.21	
	Pearson’s *r*	95% CI	Pearson’s *r*	95% CI
*PSDc_var_3Hz*	0.59	[0.55, 0.62]	0.22	[0.16, 0.27]
*PSDc_var_3Hz_log*	0.72	[0.69, 0.75]	0.43	[0.38, 0.48]
*f_tQ_mod*	−0.37	[−0.42, −0.32]	−0.31	[−0.36, −0.26]
*abs_f_tQ_dist_5Hz*	−0.44	[−0.49, −0.39]	−0.39	[−0.44, −0.34]
*avg_tQ_max*	0.67	[0.64, 0.71]	0.36	[0.30, 0.41]
*avg_tQ_max_log*	0.73	[0.71, 0.76]	0.52	[0.47, 0.56]
*sd_tQ_max*	0.60	[0.56, 0.64]	0.29	[0.24, 0.34]
*sd_tQ_max_log*	0.68	[0.65, 0.71]	0.47	[0.42, 0.51]
*cv_tQ_max*	0.18	[0.12, 0.23]	0.08	[0.02, 0.14]
*avg_alpha*	0.15	[0.09, 0.21]	0.04 ^†^	[−0.02, 0.10]
*sd_alpha*	0.27	[0.21, 0.32]	0.11	[0.05, 0.17]
*cv_alpha*	−0.32	[−0.37, −0.27]	−0.13	[−0.19, −0.07]
*avg_abs_Vt.LP.1*	−0.10	[−0.16, −0.04]	−0.08	[−0.14, −0.02]
*sd_abs_Vt.LP.1*	−0.02^†^	[−0.08, 0.04]	−0.04 ^†^	[−0.10, 0.02]
*sd_abs_Vt.LP.1_log*	0.01^†^	[−0.05, 0.07]	0.01 ^†^	[−0.05, 0.07]
*cv_abs_Vt.LP.1*	0.20	[0.14, 0.26]	0.12	[0.06, 0.18]
*avg_press*	−0.10	[−0.16, −0.04]	−0.05 ^†^	[−0.11, 0.01]
*sd_press*	0.32	[0.27, 0.37]	0.16	[0.10, 0.22]
*cv_press*	0.26	[0.21, 0.32]	0.12	[0.06, 0.18]
*Vt.HP.C5_MSEn_2_5*	0.19	[0.13, 0.25]	0.25	[0.19, 0.30]
*Vt.HP.C5_MSEn_2_10*	−0.09	[−0.14, −0.03]	0.02^†^	[−0.04, 0.08]
*Vt.HP.C5_MSEn_2_25*	−0.62	[−0.65, −0.58]	−0.42	[−0.47, −0.37]
*Vt.HP.C5_MSEn_2_50*	−0.69	[−0.72, −0.66]	−0.43	[−0.47, −0.38]

CI—confidence interval; ^†^—the 95% CI of correlation coefficient includes 0.

**Table 4:** Summary of Pearson’s correlations of model predictions and target values.

Scale	Model type	*λ_LASSO_*	Maximum	Median	Minimum	Max.–Min.
***tremor_score***	$M_{ESN}$	*λ*_1*SE*_	0.853	0.795	0.366	0.487
		*λ_MIN_*	0.854	0.794	0.388	0.466
	$M_{ESN+TS}$	*λ*_1*SE*_	0.891	0.859	0.739	0.152
		*λ_MIN_*	0.897	0.865	0.737	0.160
	$M_{TS}$	*λ*_1*SE*_	0.879	0.851	0.797	0.082
		*λ_MIN_*	0.886	0.861	0.810	0.076
	$M_{ESN}$ ensemble	*λ*_1*SE*_	0.848	0.826	0.782	0.066
		*λ_MIN_*	0.851	0.830	0.786	0.065
	$M_{ESN+TS}$ ensemble	*λ*_1*SE*_	0.884	0.865	0.827	0.057
		*λ_MIN_*	0.890	0.874	0.839	0.051
**UPDRS.21**	$M_{ESN}$	*λ*_1*SE*_	0.629	0.528	0.125	0.504
		*λ_MIN_*	0.637	0.526	0.144	0.493
	$M_{ESN+TS}$	*λ*_1*SE*_	0.638	0.571	0.487	0.151
		*λ_MIN_*	0.638	0.570	0.469	0.169
	$M_{TS}$	*λ*_1*SE*_	0.610	0.566	0.514	0.096
		*λ_MIN_*	0.628	0.574	0.522	0.106
	$M_{ESN}$ ensemble	*λ*_1*SE*_	0.610	0.551	0.505	0.105
		*λ_MIN_*	0.614	0.558	0.508	0.106
	*M_ESN+TS_* ensemble	*λ*_1*SE*_	0.626	0.578	0.527	0.099
		*λ_MIN_*	0.626	0.581	0.530	0.096
